# ATM Suppresses SATB1-Induced Malignant Progression in Breast Epithelial Cells

**DOI:** 10.1371/journal.pone.0051786

**Published:** 2012-12-10

**Authors:** Ellen Ordinario, Hye-Jung Han, Saori Furuta, Laura M. Heiser, Lakshmi R. Jakkula, Francis Rodier, Paul T. Spellman, Judith Campisi, Joe W. Gray, Mina J. Bissell, Yoshinori Kohwi, Terumi Kohwi-Shigematsu

**Affiliations:** 1 Life Sciences Division, Lawrence Berkeley National Laboratory, University of California, Berkeley, California, United States of America; 2 Buck Institute for Research on Aging, Novato, California, United States of America; University of Chicago, United States of America

## Abstract

SATB1 drives metastasis when expressed in breast tumor cells by radically reprogramming gene expression. Here, we show that SATB1 also has an oncogenic activity to transform certain non-malignant breast epithelial cell lines. We studied the non-malignant MCF10A cell line, which is used widely in the literature. We obtained aliquots from two different sources (here we refer to them as MCF10A-1 and MCF10A-2), but found them to be surprisingly dissimilar in their responses to oncogenic activity of SATB1. Ectopic expression of SATB1 in MCF10A-1 induced tumor-like morphology in three-dimensional cultures, led to tumor formation in immunocompromised mice, and when injected into tail veins, led to lung metastasis. The number of metastases correlated positively with the level of SATB1 expression. In contrast, SATB1 expression in MCF10A-2 did not lead to any of these outcomes. Yet DNA copy-number analysis revealed that MCF10A-1 is indistinguishable genetically from MCF10A-2. However, gene expression profiling analysis revealed that these cell lines have significantly divergent signatures for the expression of genes involved in oncogenesis, including cell cycle regulation and signal transduction. Above all, the early DNA damage-response kinase, ATM, was greatly reduced in MCF10A-1 cells compared to MCF10A-2 cells. We found the reason for reduction to be phenotypic drift due to long-term cultivation of MCF10A. ATM knockdown in MCF10A-2 and two other non-malignant breast epithelial cell lines, 184A1 and 184B4, enabled SATB1 to induce malignant phenotypes similar to that observed for MCF10A-1. These data indicate a novel role for ATM as a suppressor of SATB1-induced malignancy in breast epithelial cells, but also raise a cautionary note that phenotypic drift could lead to dramatically different functional outcomes.

## Introduction

Normal breast epithelial cells acquire malignant phenotypes through multiple genomic and microenvironmental modifications [1]–[3]. Metastatic and highly invasive phenotypes are often fatal. Understanding these steps is essential to treating the metastatic tumors [4].

SATB1, which functions as a genome organizer, plays a pivotal role in promoting breast tumor progression towards metastasis [5]. SATB1 is a nuclear protein that specifically recognizes and binds specialized genomic sequences that readily form a continuously unpaired structure when placed under negative super-helical strain [6]–[8]. SATB1 binds these sequences, termed base-unpairing regions (BURs), in the double-stranded DNA form. BURs of specific gene loci are tethered to a protein complex comprising SATB1, which then serves as an architectural platform to recruit transcriptional regulators and chromatin remodeling proteins to alter the epigenetic states of target gene loci [9], [10]. SATB1, thereby, regulates a large number of genes by folding chromatin into loops [11] and promotes growth and metastasis of breast tumors by reprogramming chromatin organization and altering the transcription of up to 1000 genes [5]. This ‘genome organizing’ activity of SATB1 is critical for changes in cellular functions such as T cell differentiation [12], [13], T cell activation [11], postnatal cortical development [14], X-chromosome inactivation [15], epidermal differentiation [16] and progression of breast tumors to metastasis [5].

SATB1 is expressed in a number of aggressive cancer cell lines and poorly differentiated human breast tumor biopsies [5], [17]–[20]. In contrast, SATB1 expression is either very low or undetectable in non-aggressive cancer cell lines and normal mammary epithelial cells [5]. Forced expression of SATB1 in breast cancer cell lines, such as SKBR3, converts them to aggressive tumor cells. Conversely, silencing SATB1 expression by RNA interference in highly metastatic human breast cancer cell lines, such as MDA-MB-231 and BT549, abolishes their ability not only to metastasize, but also to form tumors in mice [5]. Immunostaining of tissue arrays containing ∼1000 human breast cancer biopsies showed that high nuclear SATB1 staining correlated with patients' poor prognosis (P<0.0001) [5]. These observations suggest that deregulation of SATB1 in malignant cells alone, in lieu of multiple successive genomic aberrations, is sufficient to alter the expression of a large number of genes required for progression of cancer to metastasis [5].

Involvement of SATB1 in breast cancer has been shown also by independent studies [17], [19]–[22]. Furthermore, recent reports have expanded the association of SATB1 with multiple types of tumors in addition to breast cancer, such as laryngeal squamous cell carcinoma, endometriod endometrial cancer, hepatocellular carcinoma, rectal cancer, cutaneous malignant melanoma, and gastric cancer [21]. There have been two reports that do not observe a correlation between expression of SATB1 mRNA and breast malignancy [23], [24]. However, to assess SATB1 presence in breast tumor specimens, it is crucial to examine individual tumor cells by immunohistochemistry rather than by total mRNA isolated from whole tissues because in some tumor specimens, SATB1 is expressed in surrounding stromal cells as well [21]. By immunohistochemical analyses, other studies have shown that high SATB1 expression correlates with metastasis of cutaneous malignant melanoma and gastric cancer [25]–[27], a finding consistent with SATB1's role in breast cancer metastasis [5].

Whereas earlier studies examined SATB1's role in the progression of tumor cells, here we took a more direct approach and asked whether forced expression of SATB1 in non-malignant breast epithelial cells could induce a malignant phenotype. The non-tumorigenic breast epithelial cell line, MCF10A [28], is used widely to characterize cellular processes involved in the early stages of tumorigenesis, such as proliferation, migration and morphogenesis [29]–[32]. Because cell lines often change function in culture, we obtained aliquots of MCF10A from two different sources, which we refer to as MCF10A-1 and MCF10A-2 (see Materials and Methods). We show that ectopic expression of SATB1 converted MCF10A-1, but not MCF10A-2, to full-blown tumorigenicity including metastases to lungs. Gene expression analysis of MCF10A-1 and MCF10A-2 revealed different expression patterns of genes involved in cell cycle regulation and identified the level of the early DNA damage-response kinase, ATM (ataxia telangiectasia mutated), as a potential reason behind the differential response to SATB1 expression. Forced SATB1 expression in MCF10A-2 cells, as well as in the non-tumorigenic breast epithelial cell lines 184A1 and 184B5 [33]–[35] that express higher levels of ATM than MCF10A-1 cells, did not alter their cellular phenotypes. Strikingly however, reduction of ATM levels in these same cells allowed them to acquire a malignant phenotype. These results uncover a tumor suppressive function of ATM in breast tumorigenesis and metastasis, and suggest that *ATM* down regulation is critical for the oncogenic activity of SATB1. The results also draw fresh attention to the universal observation of phenotypic drift in cultured cells [36] and the necessity of examining multiple sources of a given cell line before drawing cause and effect conclusions with cultured cells.

## Results

**Figure 1:**
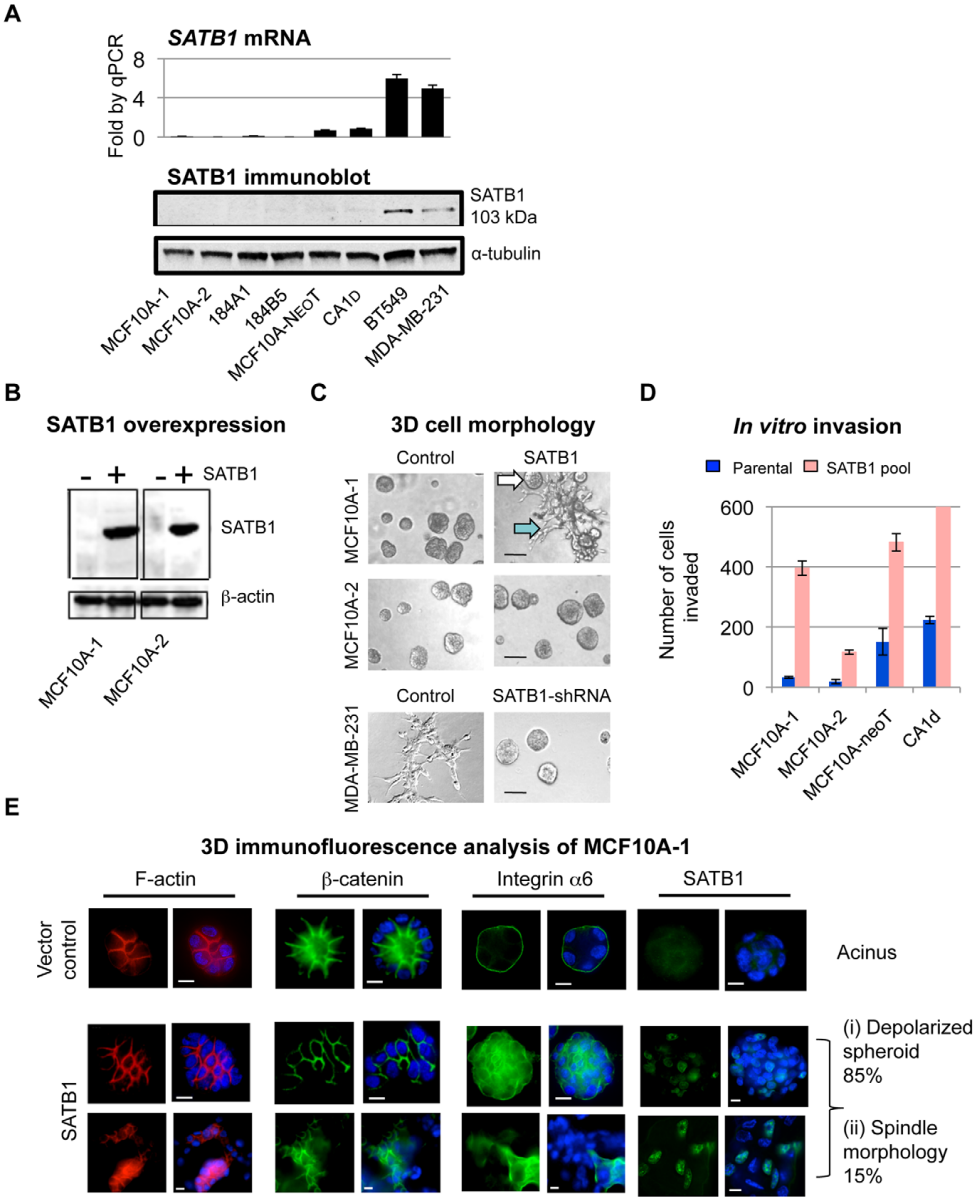
SATB1 is endogenously expressed in aggressive breast cancer cell lines and its ectopic expression induces malignant phenotype in non-malignant MCF10A-1, but not in MCF10A-2 cells. **A**) (Top) Quantitative RT-PCR analysis for SATB1 expression relative to GAPDH in MCF10A cell lines from two different sources (MCF10A-1 and MCF10A-2), 184A1, 184B5 and MCF10A progression series (MCF10A-neoT and CA1d), BT549 and MDA-MB-231. (Bottom) Immunoblot for SATB1 expression using the same cell lines as in quantitative RT-PCR analysis. α-tubulin was used as an internal loading control. **B**) Immunoblot showing the expression level of SATB1 before and after SATB1 overexpression in MCF10A-1 and MCF10A-2 cells. ß-actin was used as an internal loading control. **C**) Colony morphologies of MCF10A-1 and MCF10A-2 control and SATB1 overexpressing cells (pLXSN-SATB1: pooled populations), and MDA-MB-231 control and SATB1 depleted (SATB1 shRNA) cells at six days of 3D matrix on-top culturing. Images were captured with Phase 1 at 20X magnification. Scale bars, 100 µm. Note that SATB1 overexpression caused MCF10A-1 to form a mixture of large spheroid and spindle structures, indicated by white and blue arrows, respectively, while it caused MCF10A-2 cells to form larger spheroid structures. The aggressive breast cancer cell line, MDA-MB-231, formed network-like spindle structures, which was inhibited by knockdown of SATB1. **D**) Invasion assay of MCF10A-1, MCF10A-2, MCF10A-neoT and CA1d cell lines before and after SATB1 overexpression. Parental cells lines are shown in blue; SATB1 overexpressing cells are shown in pink. Error bars indicate s.e.m., n = 3 experiments. **E**) Vector control (top row) and SATB1-expressing MCF10A-1 (pLXN-SATB1) (bottom two rows) grown on Matrigel were stained for F-actin (red), ß-catenin (green), integrin α6 (green), SATB1 (green) and DAPI (blue). Note that control cells showed the typical acinar structure whereas SATB1-expressing cells showed large spheroid (i) and spindle (ii) structures. No acinar structures were detected in SATB1-expressing MCF10A-1. Scale bars, 15 µm.

SATB1 expression was shown previously to promote metastasis in established but non-metastatic breast cancer cells [5]. To address whether SATB1 is able also to induce malignancy in non-tumorigenic cells, we examined SATB1 levels in eight non-tumorigenic and tumorigenic breast epithelial cell lines. The non-tumorigenic cell line analyzed, MCF10A, was established after spontaneous immortalization of primary human mammary epithelial cells derived from reduction mammoplasty [28], [37], [38]. We examined two different sources of these cells, designated MCF10A-1 and MCF10A-2 and two other immortalized non-malignant cell lines, 184A1 and 184B5 that were derived from reduction mammoplasty of a normal human breast epithelial cells (specimen#184) after treatment with benzo(a)pyrene [33]–[35]. In addition, we analyzed the MCF10A cancer progression series: premalignant neoT cells (also called MCF10AT; [39]) expressing mutant *HRAS* and malignant Ca1d (undifferentiated carcinoma with low metastatic potential) [39]–[41]. Quantitative RT-PCR and immunoblot analyses showed that endogenous SATB1 levels were very low to undetectable in all immortalized non-tumorigenic cell lines tested in contrast to its easily detectable expression in metastatic human breast cancer cell lines, MDA-MB-231 and BT549 (Fig. 1A). Endogenous SATB1 expression levels (for both mRNA and protein) in cells from the MCF10A progression series were detectable, but significantly lower- 1/6^th^ to 1/7^th^- than those in aggressive cancer cell lines (Fig. 1A).

To determine whether ectopic SATB1 expression induces a malignant phenotype in non-tumorigenic cells, we transduced MCF10A-1 and MCF10A-2 cells with a retroviral vector to overexpress SATB1 (pLXSN-SATB1) (Fig. 1B). We also overexpressed SATB1 in neoT and Ca1d of the MCF10A cancer progression series (Fig. S1A) and examined the colony morphology in three dimensional (3D) cultures grown on top of Matrigel (Fig. 1C and Fig. S1B). Upon SATB1 overexpression, neoT and Ca1d cells underwent major morphological changes from spherical to spindle-shaped network structures (Fig. S1B). These altered colony morphologies resembled the structures formed by aggressive breast cancer cell lines, MDA-MB-231 and BT549, which express a high level of endogenous SATB1 (Fig. 1C; Fig. S1B, right). shRNA-mediated SATB1 knockdown in MDA-MB-231 and BT549 cells reversed the colony morphologies from stellate to spherical structures as reported previously (Fig. 1C; Fig. S1B, right) [5], suggesting a direct role of SATB1 in the stellate morphology in these cells. Interestingly, SATB1 overexpression also altered the colony morphology of MCF10A-1, from uniformly spherical to a mixture of spherical (white arrow) and spindle-shaped structures (blue arrow), representing 85% and 15% of the cell population, respectively (Fig. 1C). On the other hand, SATB1 overexpression in another MCF10A-2, only led to the formation of slightly enlarged spheres (Fig. 1C). This result suggested that these two MCF10A cultures possess different properties responsible for their differential responsiveness to SATB1 overexpression.

The abnormal 3D morphology of SATB1 expressing MCF10A-1 cells led us to ask whether ectopic SATB1 expression induces invasive properties in these cells. Invasion assays demonstrated that SATB1 overexpression elevated the invasive potential of multiple cell lines derived from MCF10A cells compared to the parental cell lines. For MCF10A-1 and neoT cells, the invasiveness increased by approximately 400-fold (Fig. 1D). Ca1d cells showed the highest increase (more than 800-fold), while MCF10A-2 cells showed the least increase (Fig. 1D). To examine whether the altered 3D morphology upon SATB1 expression reflected changes in cell polarity, we immunostained MCF10A-1 cells expressing control vector or SATB1 (pLSXN-SATB1) for filamentous actin (F-actin), ß-catenin and α6-integrin (Fig. 1E). These proteins are markers for properly formed mammary acini, which are glandular-like structures with a hollow lumen surrounded by polarized epithelial cells [42]. In 3D cultures, MCF10A cells form acinar structures that have basal polarity with a hollow center resembling a lumen [43]–[45], as evidenced by staining of control MCF10A-1 cells (Fig. 1E, top row). Immunostaining of SATB1-expressing MCF10A-1 cells revealed that the spherical colonies had lost cell basal polarity, as indicated by reduced ß-catenin expression and disorganized F-actin and α6- integrin, and did not posses hollow lumen (Fig. 1E, center row). Spindle-shaped colonies exhibited even more aberrant localization of these markers (Fig. 1C, blue arrow; Fig. 1E, bottom row). To examine the level of SATB1 expression in the spherical and spindle colonies, we immunostained SATB1-expressing MCF10A-1 cells for SATB1 on the 6^th^ day of 3D culturing (Fig. 1E, far right). The results showed that SATB1 expression was found in both the depolarized spheroids and spindle- shaped colonies, with SATB1 detected in approximately 50% of the cells in a single colony. In comparison to the spherical colonies, the spindle colonies appeared to have a greater signal intensity for SATB1.

**Figure 2:**
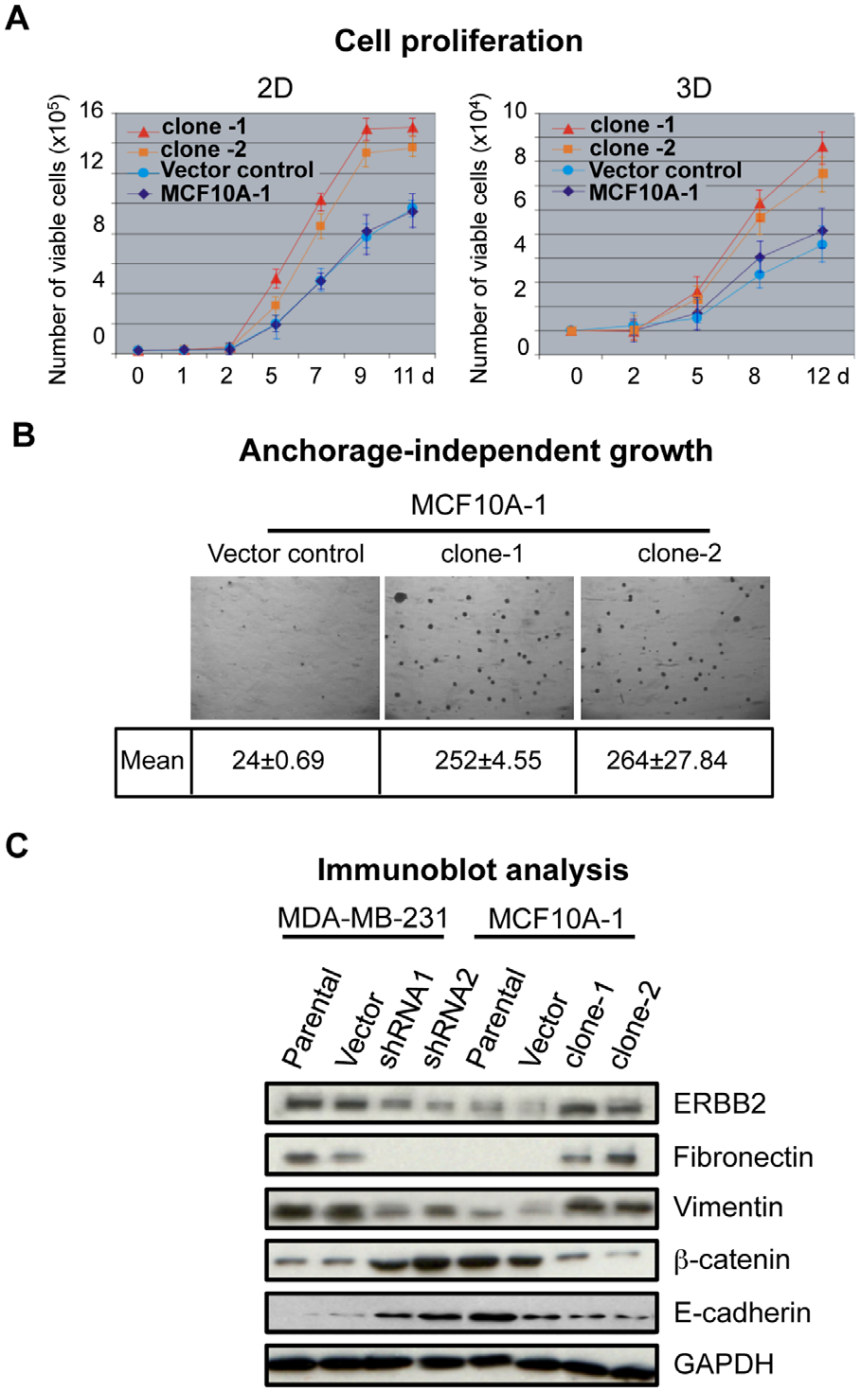
SATB1 overexpression induces EMT in MCF10A-1 cells. **A**) Cell proliferation assay to compare the growth over time (days, d) of the parental MCF10A-1 and vector control cells with single-cell-derived SATB1-overexpressing MCF10A-1 clones clone-1 and clone-2 on plastic dishes (2D) or on Matrigel (3D). Error bars indicate ±s.e.m. from three independent experiments. **B**) Representative photographs of soft agar colonies formed by control and SATB1 overexpressing (clone-1 and clone-2) MCF10A-1 cells after 25 days of culture. The mean colony counts from three replicates are shown. **C**) Immunoblot analyses for the expression of mesenchymal markers (fibronectin and vimentin), epithelial markers (E-cadherin and ß-catenin) as well as SATB1 target ERBB2 in MDA-MB-231 (parental, control and SATB1-depleted by shRNA1 or shRNA2) and MCF10A-1 (parental, control, clone-1 and clone-2). Cell lysates were prepared from cells cultured on plastic dishes (2D). GAPDH was used as a loading control.

To validate the observed changes caused by SATB1 overexpression in MCF10A-1 cells, we isolated two single-cell-derived clones, clone-1 and clone-2, which expressed SATB1 at high levels from pooled population (Fig. S1C). Compared to the parental and vector control populations, clone-1 and clone-2 cells proliferated at significantly higher rates, whether on plastic in 2D or on top 3D gels (Fig. 2A). Morphological analysis of both clones in 3D cultures showed predominantly spindle colonies with a few spherical colonies (Fig. S1C). In soft agar, both SATB1-overexpressing clones formed colonies with larger size and approximately 10-fold higher numbers over those formed by the vector control (Fig. 2B).

The SATB1-induced changes in colony morphology; invasive potential and anchorage-independent growth all support its role in driving epithelial-to-mesenchyme transition (EMT). EMT entails loss of epithelial cell polarity, cell-to-cell contact and cytoskeletal organization, and presages the development of aggressive phenotypes during cancer progression [46]. EMT is characterized by upregulation of mesenchymal markers, fibronectin and vimentin, and loss of an epithelial marker, E-cadherin. To test if SATB1 overexpression indeed alters the expression of these EMT markers in MCF10A1 cells, we examined the levels of these proteins in MCF10A-1 derivatives including parental cells, control vector, clone-1 and clone-2 by immunoblot analysis. As positive control cell lines, we used MDA-MB231 cells to express each of two SATB1 shRNAs (shRNA1, shRNA2) [5] (Fig. 2C). SATB1 is known to directly regulate *ERBB2*, an important regulator of breast cancer progression [47], in MDA-MB-231 cells [5]. Therefore in this assay, we used ERBB2 as a positive control: SATB1 expression in MCF10A-1 cells increased ERBB2 expression, whereas depletion in MDA-MB-231 cells greatly decreased ERBB2 expression. SATB1 overexpression in MCF10A-1 cells also led to an increase in fibronectin and vimentin. Conversely, SATB1 depletion in aggressive MDA-MB-231 cells led to a loss or reduction of these proteins (Fig. 2C). In particular, fibronectin, at the protein level, appeared to be entirely dependent on SATB1 expression; while at the transcript level, it was shown to be reduced only 2-fold in MDA-MB-231 upon SATB1 knockdown [5] (Fig. 2C). In contrast to fibronectin, ß-catenin levels were greatly reduced and E-cadherin levels were slightly decreased upon SATB1 overexpression in MCF10A-1 cells (Fig. 2C). In conjunction with the immunoblot analysis, immunostaining experiments in clone-1 confirmed the SATB1-dependent regulation of fibronectin and ß-catenin. (Fig. S1D). These results suggest that SATB1 expression induces EMT and causes a gain of cancer phenotypes in non-malignant cells.

**Figure 3:**
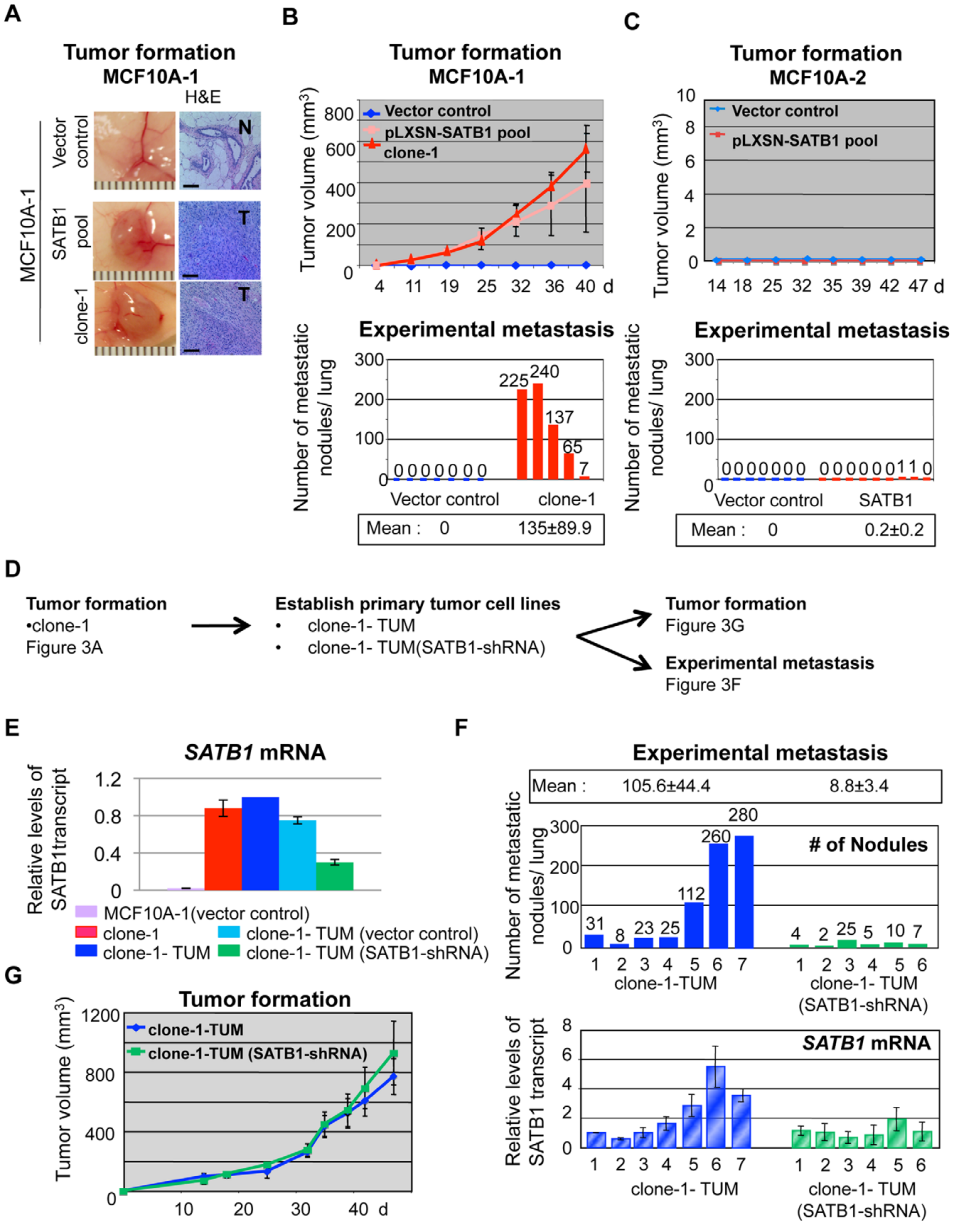
Ectopic expression of SATB1 in MCF10A-1 cells induces tumor growth and lung colonization. **A**) (Left) Representative photographs of tumors formed by vector control and SATB1 overexpressing (pooled population and clone-1) MCF10A-1 cells injected into the mammary fat pad of nude mice. (Right) Corresponding tumor sections stained with haematoxylin and eosin. T, tumor; N, normal breast tissue. Scale bar, 80 µm. **B**) (Top) Mean tumor volumes as formed in (**A**). Note that the tumors derived from clone-1 grew at the fastest rate. Each data point is shown as the mean value (±s.e.m.) of 8–10 primary tumors. (Bottom) The number of metastatic nodules per lung formed by MCF10A-1 vector control (n = 7) and clone-1 (n = 5) 9 weeks after injection into the tail vein. The mean values for each group are underlined and shown below plot. **C**) (Top) The growth of tumor by vector control and SATB1 overexpressing (pLXSN-SATB1) MCF10A-2 cells after injection into mammary fat pads of nude mice. Note that MCF10A-2 cells did not form tumors upon SATB1 expression. (Bottom) The number of metastatic nodules per lung formed by MCF10A-2 vector control (n = 7) and SATB1 pool (n = 9) 9 weeks after injection into the tail vein. The mean values for each group are underlined and shown below plot. **D**) Schematic diagram for tumor formation and metastasis experiments. Primary tumor cell lines clone-1-TUM derived from xenografts of SATB1 overexpressing MCF10A-1 clone-1 were transfected with SATB1-shRNA and used for *in vivo* tumor formation and experimental metastasis experiments. **E**) Quantitative RT-PCR analysis for SATB1 transcript levels in parental MCF10A-1, clone-1, clone-1-TUM, clone-1-TUM (vector control), and clone-1-TUM (SATB1-shRNA) cells relative to GAPDH. **F**) (Top) The number of metastatic nodules per lung by the pooled population of clone-1-TUM (n = 7) and clone-1-TUM (SATB1-shRNA) (n = 6) 9 weeks after the injection into the tail vein of nude mice. The mean nodule per lung is underlined and shown above plot. (Bottom) The level of human SATB1 expression in mouse lungs relative to actin. RNAs were prepared from the metastatic lung of each injected nude mouse as described in (Top). Quantitative RT-PCR analysis was performed to evaluate the human specific SATB1 expression originating from the injected cells. The relative levels of SATB1 expression against sample #1 of clone-1-TUM (indicated as one) are shown. **G**) The growth of tumor formed by parental or SATB1-shRNA clone-1-TUM cells injected into mammary fat pads of nude mice. Mean volumes (n = 6 per group) of tumors formed in fat pads of mice are shown.

To verify the observations made in cell cultures, we tested if SATB1-expressing MCF10A-1 and MCF10A-2 cells could form tumors in nude mice. We injected SATB1-overexpressing MCF10A-1 cells (pooled pLXSN-SATB1 and single cell-derived clone-1) or vector control cells into the fat pad of the fourth mammary gland and monitored tumor formation. Both types of SATB1-overexpressing MCF10A-1 cells formed large tumors in all the injected mice, whereas control cells did not (Figs. 3A, 3B, and Fig. S2A). Furthermore, to test whether SATB1-overexpressing MCF10A-1 cells exhibit elevated migration and invasion properties *in vivo*, we injected clone-1 or control cells into the tail veins of nude mice and scored metastatic nodules in the lungs (Fig. 3B, lower panel). A large number of metastatic nodules (average = 135±89.9/lung) were formed from clone-1 cells (4/5 mice injected) while none were formed from control (Fig. 3B, lower panel). These data demonstrated that SATB1 overexpression in MCF10A-1 induces cells to adopt a metastatic cancer phenotype *in vivo*. In contrast to MCF10A-1, *in vivo* analysis showed that MCF10A-2 cells, which did not exhibit a pronounced change in their 3D morphology (Fig. 1C) and invasiveness (Fig. 1D) upon SATB1 overexpression, failed to form tumors in nude mice (Fig. 3C) and did not produce an appreciable number of metastatic nodules (Fig. 3C, lower panel).

We examined whether the maintenance of the aggressive phenotype in MCF10A-derived tumor cells requires sustained SATB1 expression. As illustrated in Fig. 3D, we isolated clone-1-TUM cells from tumors derived from xenografts of SATB1-expressing MCF10A-1 clone-1 (Fig. 3A). We knocked down SATB1 expression from clone-1-TUM cells by shRNA [clone-1-TUM (SATB1-shRNA)] and reduced the overall SATB1 transcript level by ∼70% compared to control cells (Fig. 3E). The residual SATB1 expression after knockdown could possibly be due to the varied knockdown efficacies of SATB1-shRNA for different cells within the clone-1-TUM population. We then injected clone-1-TUM or clone-1-TUM (SATB1-shRNA) cells into the tail veins of mice (Fig. 3F, upper panel). Of 7 mice injected with clone-1-TUM cells, 3 mice had >100 nodules and the remaining 4 mice had 8–31 nodules each, with an average of 105 nodules per lung. In contrast, clone-1-TUM (SATB1-shRNA) cells, which still retain 30% SATB1 expression, produced far fewer metastases. Of 6 mice, all had 25 or fewer nodules, with an average of 9 nodules per lung (Fig. 3F, upper panel). These data indicate that the aggressive phenotype of clone-1-TUM is reversible by reducing SATB1 levels.

We observed varying degrees of lung metastases by clone-1-TUM cells, which may be attributable to different SATB1 expression levels. To address this possibility, we isolated the metastatic lung tissues and performed quantitative RT-PCR. The levels of human SATB1 transcripts, normalized to human actin levels, showed a positive, although not directly linear, correlation between the levels of SATB1 mRNA and the number of metastatic nodules per lung (Fig. 3F, lower panel). These results support the contention that high SATB1 levels drive MCF10A-1 cells to form lung metastases. In contrast to lung metastasis, when we examined tumor formation by injecting clone-1-TUM or clone-1-TUM (SATB1-shRNA) into the mammary fat pads of nude mice, we found virtually no difference in the ability to form tumors by these two cell lines (Fig. 3G), except for a slight delay in onset of tumor growth by the latter during the first 2 weeks (Fig. S2B). These *in vivo* data indicate that the SATB1 expression level is critical for tumor progression; whereas low levels of SATB1 expression are sufficient for tumor formation, higher levels of SATB1 are necessary for lung metastasis of MCF10A-1 cells.

**Figure 4:**
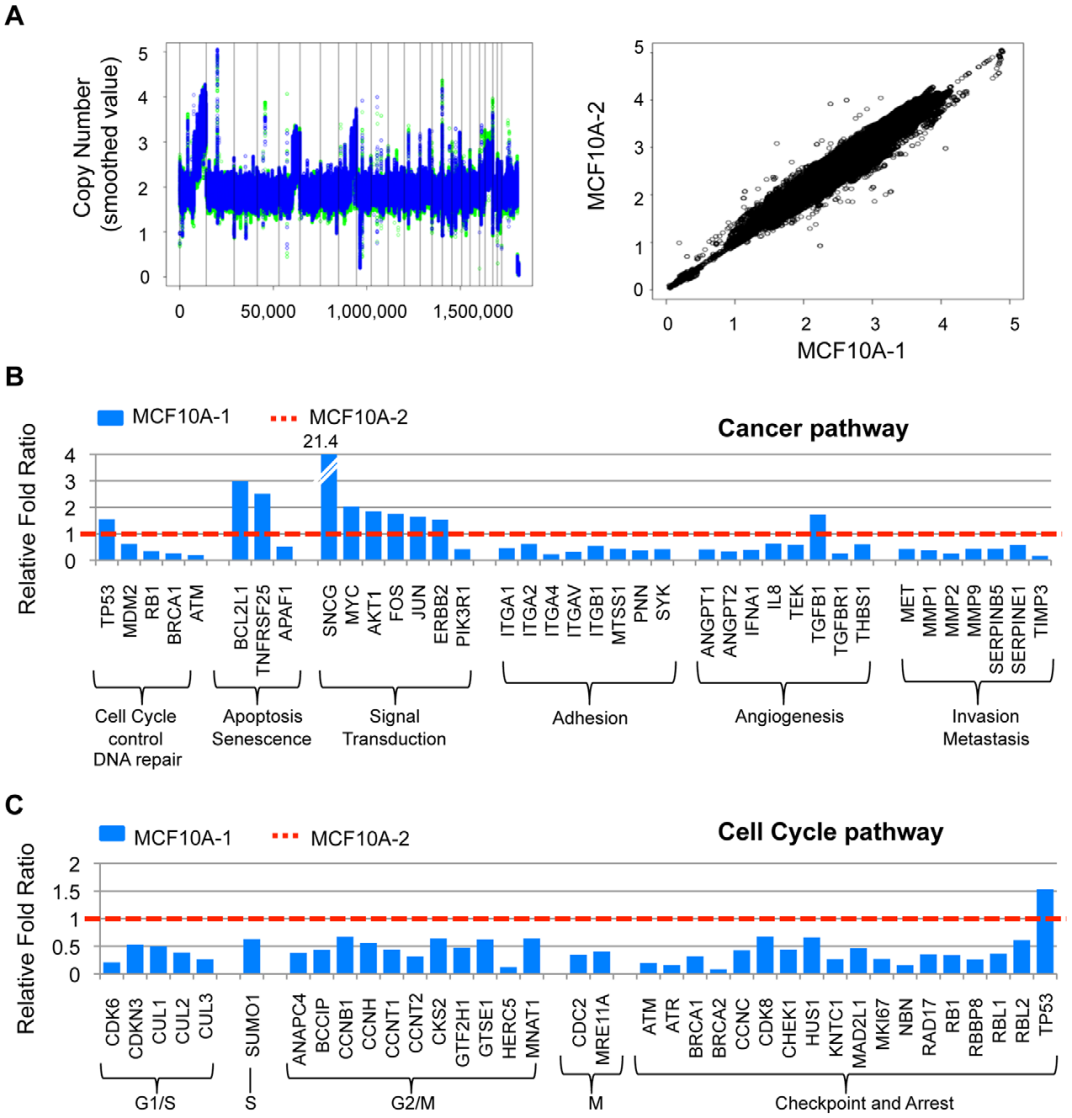
Differential response to SATB1 overexpression by MCF10A-1 and MCF10A-2 cells is attributed to their disparate gene expression patterns involved in cell cycle regulation. **A**) (Left) Gene copy number profiles for MCF10A-1 and MCF10A-2. Each dot represents the copy number for a given SNP that is ordered by genomic position, from chromosome 1 to chromosomes X and Y. Vertical lines represent chromosome boundaries for the autosomes. Data for MCF10A-1 and MCF10A-2 are plotted in green and blue, respectively. The copy number profiles for both cell lines were largely overlapping, indicating that the cell lines are genomically the same. (Right) Copy number correlation between MCF10A-1 and MCF10A-2. Each dot represents copy number for a single SNP, where copy number for the MCF10A-1 cell line is plotted along the x-axis and that for the MCF10A-2 cell line is plotted along the y-axis. The Pearson correlation is 0.96 (p<2.2e-16), which supports the idea that the two cell lines are identical. **B**) Expression analysis of genes associated with cancer progression in MCF10A-1 and MCF10A-2. Genes are categorized based on their specific biochemical functions. Fold gene expression in MCF10A-1 (blue bar) relative to MCF10A-2 (red dotted line at 1 fold) is shown. Each gene expression level was normalized to the level of GAPDH. **C**) Expression analysis of genes associated with cell cycle regulation in MCF10A-1 and MCF10A-2. Genes are categorized based on their roles in specific cell cycle phases. The result is shown as in (**B**).

The contrasting behaviors of MCF10A-1 and MCF10A-2 upon SATB1 expression highlighted the possibility that fundamental differences in the genomes of the two cell lines may be responsible for the disparate responsiveness to SATB1 activity. To determine whether MCF10A-1 and MCF10A-2 exhibit a gross genotypic difference, we performed genome-wide copy number analysis to compare genomes (Fig. 4A). The genotypes of the MCF10A-1 and MCF10A-2 were 98.83% identical, providing strong evidence that they were derived from the same predecessor. Furthermore, analyses of their copy number profiles revealed that they were very similar as determined by a Pearson correlation of 0.96 (p<2.2e-16), providing additional evidence that these two lines are genomically identical. It has been reported that the copy number profiles of non-malignant MCF10A and premalignant neoT only differ in chromosome 9.13 and 9.20 [48]. We found no differences in the copy number profiles from these regions for MCF10A-1 and MCF10A-2 cells (Fig. S3A). In addition, p53 was similarly activated in MCF10A-1 and MCF10A-2 cells [49], [50], as determined by phosphorylation of serine 15 and by induction of downstream genes, p21 and MDM2, upon exposure to ionizing radiation (12 Gy X-ray) (Fig. S3B), suggesting that p53 is intact in both cell lines.

To identify the molecular bases for the contrasting phenotypes of MCF10A-1 and MCF10A-2 cells upon SATB1 overexpression, we analyzed gene expression patterns. Quantitative RT-PCR using the Cancer Pathway Superarray (89 genes) revealed differential expression (>1.5 fold up- or down-modulation with p<0.05) of genes involved in cell cycle control/DNA repair, adhesion, angiogenesis and invasion/metastasis. Notably, *ATM* mRNA levels were 10-fold lower in MCF10A-1 compared to MCF10A-2 cells (Fig. 4B). Additional analyses using the Cell Cycle Pathway and p53 Pathway arrays (89 genes each) confirmed that the expression of cell cycle and checkpoint genes significantly differed between the two cell lines (Fig. 4C; Fig. S3C). Among the cell cycle/checkpoint-related genes, *ATM* and *ATR* (ATM and Rad3-related) were those that were the most significantly downregulated in MCF10A-1 cells compared to MCF10A-2 cells. ATM and ATR are members of the PI3-kinase family that sense and transduce responses to DNA double strand breaks and regulate cell cycle checkpoints, DNA repair, apoptosis and senescence [51]–[53]. Both ATM and ATR share many biochemical and structural similarities, but differ in certain cellular activities. For example, ATM is critical for the mitotic checkpoint [54], [55] and its mutation is associated with human cancer [56], whereas ATR is not. We then decided to focus on ATM and explore whether the difference in the ATM level between the MCF10A-1 and MCF10A-2 accounts for their contrasting responsiveness to SATB1 overexpression.

We tested whether MCF10A-1 cells, which have reduced ATM expression, were impaired for G2/M cell cycle checkpoints. To this end, we arrested cells at the G1/S boundary of the cell cycle using hydroxyurea (HU), released cells from HU, and immediately added paclitaxel, a drug which inhibits mitosis by stabilizing microtubule polymerization and causes G2/M cell cycle blockage [57] (Fig. S4). After 18 h of incubation with paclitaxel, MCF10A-1 and MCF10A-2 cells were mostly arrested in G2/M phase. However, after 22 h of paclitaxel incubation, there was a slight increase [∼8% (p = 0.0078)] in MCF10A-1 cells that had bypassed the G2/M checkpoint and entered G1 phase compared to MCF10A-2 cells (Fig. S4). The escape from the G2/M arrest was followed by apoptosis, which occurred at a later time point (28 h) for both MCF10A-1 and MCF10A-2. These results suggest the G2/M checkpoint for MCF10A-1 cells is slightly impaired compared to MCF10A-2.

Next, we asked whether *ATM* mRNA levels are consistently reduced in SATB1-responsive cells, such as MCF10A-1 and MCF10-neoT, which both could be induced to become malignant by SATB1 overexpression, compared to SATB1-resistant cells, such as MCF10A-2. Indeed, *ATM* mRNA was lower in MCF10A-1 and in several other SATB1-responsive breast cancer cell lines as compared to MCF10A-2 cells (Fig. S5A), suggesting that the level of ATM negatively correlates with the responsiveness to SATB1 overexpression. We found that the ATM protein and transcript levels were comparable for MCF10A-1 cells and MCF10A-2 cells at early passage, but declined after continuous passage in culture for 90 days. This observation indicates that during passage ATM expression shifts from high to low in MCF10A cells, leading to a phenotypic drift from SATB1-resistant to SATB1-responsive types (Fig. S5A and S5B). The result also suggests that MCF10A-1 cells could be derived from MCF10A-2 cells and that they are not independent sublines. Prolonged continuous culture also induced SATB1 expression at a low, but detectable, level (Fig. S5C). These results suggest that MCF10A-1 is not a rare variant of MCF10A cells and that MCF10A cells are prone to downregulate ATM upon prolonged culture and acquire phenotypes exhibited by MCF10A-1 cells. Given that early passage MCF10A-1 express ATM at levels comparable to MCF10A-2 (Fig. S5A), we tested whether early passage MCF10A-1 behave similarly to MCF10A-2 upon SATB1 expression. We overexpressed SATB1 in early passage MCF10A-1 and analyzed 3D morphology (Fig. S5D). After 6 days culture on top 3D gels, SATB1 overexpression in early passage MCF10A-1 and in MCF10A-2 both formed spherical structures and no detectable spindle colonies (Fig. S5D). *In vitro* invasion assays also demonstrated that early passage MCF10A-1 exhibited similar levels of invasive potential to MCF10A-2 upon SATB1 overexpression (Fig. S5E).

**Figure 5:**
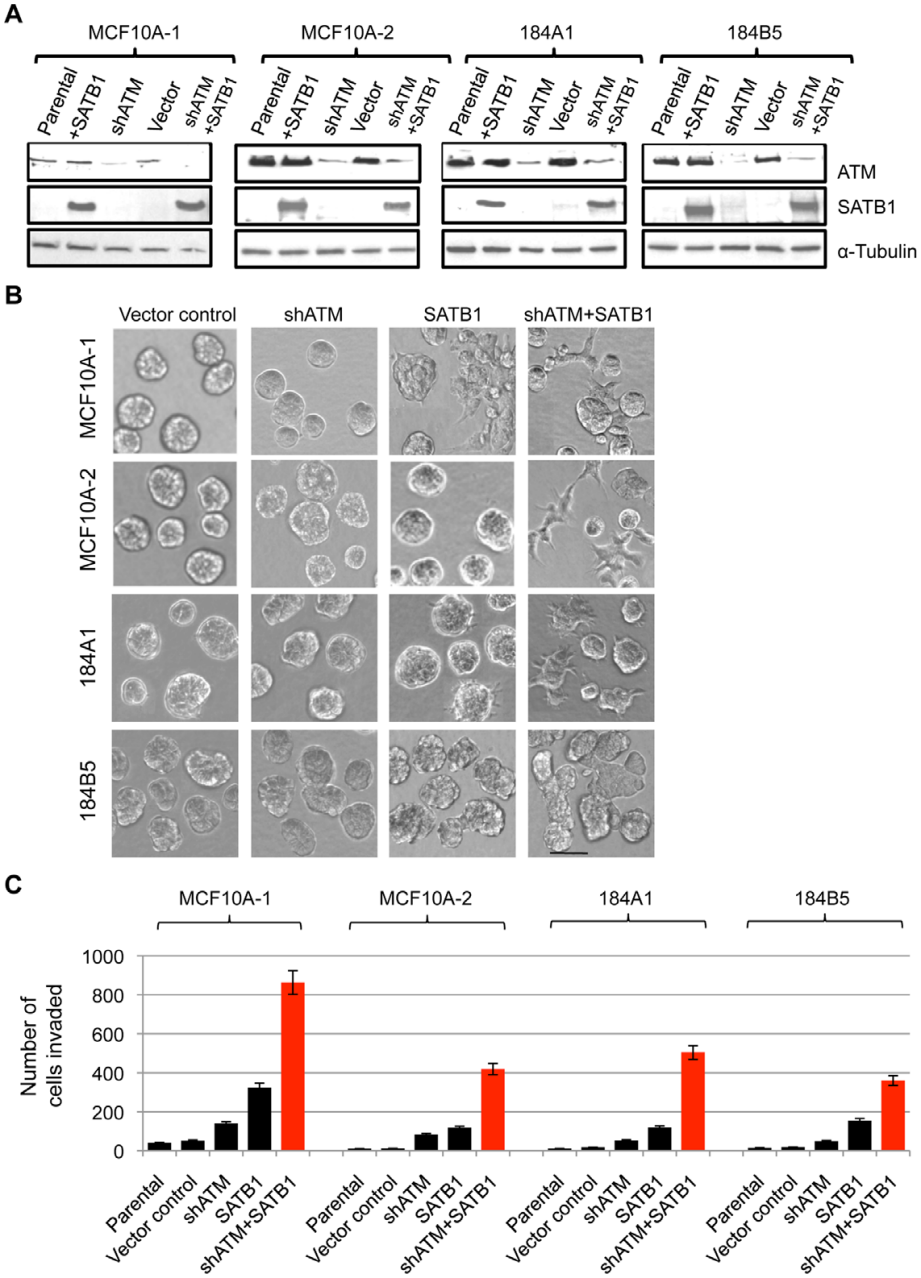
The combination of ectopic SATB1 expression and ATM depletion induces aggressive phenotype in non-malignant mammary epithelial cells. **A**) Immunoblot showing ATM and SATB1 levels before and after SATB1 overexpression (SATB1) and ATM depletion (shATM) in non-malignant MCF10A-1, MCF10A-2, 184A1 and 184B5 cells. α-tubulin was used as an internal loading control. **B**) Colony morphologies of vector control, ATM depleted (shATM), SATB1 overexpressing (SATB1) and ATM depleted/SATB1 overexpressing (shATM+SATB1) cells generated as in (**C**). Cells were grown in 3D Matrix on-top cultures. The images were captured with phase 1 at 20X magnification on five days after plating. Scale bars, 100 µm. **D**) Invasion assay of ATM depleted (shATM), SATB1 overexpressing (SATB1) and ATM depleted/SATB1 overexpressing (shATM+SATB1) cells generated as in (**a**). Error bars indicate ±s.e.m., n = 3 experiments.

We then tested whether ATM depletion is sufficient to convert MCF10A-2 cells from SATB1-resistant to SATB1-responsive types. We generated MCF10A-2 clones that stably express a shRNA against ATM (shATM) [58] together with a SATB1-overexpressing construct (Fig. 5A). Expression of the control vector or shATM alone did not alter MCF10A-2 phenotypes. However, co-expression of shATM and SATB1 induced a malignant cell morphology (Fig. 5B) and invasive activity (Fig. 5C). Thus, ATM depletion rendered MCF10A-2 cells responsive to SATB1-mediated malignant induction, suggesting a role of ATM in suppressing tumorigenic progression driven by SATB1.

To determine whether the oncogenic activity of SATB1 could be induced in other non-malignant cells lines besides MCF10A, we analyzed two additional immortalized, non-malignant human mammary epithelial cell lines, 184A1 and 184B5 [33]–[35]. 184A1 cells are stable and 184B5 cells are semi-stable during passage in culture, in contrast to MCF10A cells that are unstable and susceptible to phenotypic drifts depending on culture conditions [59], [60]. ATM levels in the two 184 cell lines were comparable to MCF10A-2 (Fig. S5A), and neither cell line adopted malignant phenotypes upon ectopic SATB1 expression. However, after stable ATM knockdown (Fig. 5A), both 184A1 and 184B5 cells became SATB1-responsive and exhibited malignant phenotypes upon SATB1-overexpression. In 3D culture, ATM knockdown and SATB1 overexpression led 184A1 cells to exhibit a spindle-like morphology and led 184B5 cells to form large irregular aggregates (Fig. 5B). Furthermore, ATM knockdown and SATB1 overexpression promoted the invasive activities of 184A1 and 184B5 cells as well as MCF10A cells (Fig. 5C). These observations further suggest that ATM serves to protect cells from transformation upon SATB1 overexpression.

## Discussion

The cell culture system has been a valuable resource that has granted researchers incredible discoveries in biology. Despite the advantages of using a cell culture-based system to elucidate biological functions, it has been long known that cells in culture undergo phenotypic drift, often giving rise to diverse experimental outcomes [36]. The molecular changes that underlie phenotypic drift, however, have been largely neglected and are not well understood. By using cell culture to study oncogenesis, one has to take into account of phenotypic drift, while carefully recreating the extracellular and intracellular environment in order to recapitulate malignant phenotypes *in vivo*. Through the use of SATB1 ectopic expression, we discovered that there are at least two populations of non-tumorigenic MCF10A cells with distinct behaviors: the SATB1-responsive cell line MCF10A-1, which could be induced by SATB1 overexpression to adopt a malignant phenotype, and the SATB1-resistant cell line MCF10A-2, which was relatively nonresponsive to SATB1's oncogenic activity and remained non-malignant after overexpression of the protein. We show here that ectopic expression of SATB1 alone is sufficient to convert MCF10A-1 cells to aggressive metastatic cancer cells both in culture and *in vivo*.

In studying the contrasting effects of SATB1 ectopic expression between MCF10A-1 and MCF10A-2, we discovered an intriguing relationship between SATB1 and ATM. We found that the oncogenic activity of SATB1 is dependent on the level of ATM expression in these immortalized cell lines. We compared MCF10A-1 and MCF10A-2 by genome-wide copy number analysis for the global genomic structure and found that both cell lines showed no significant differences. We did, however, find significant differences in the expression of genes involved in carcinogenesis, including cell cycle-related, signal transduction, and invasion/metastasis genes. These genes have crucial roles in the DNA damage-response and, therefore, in the propensity for acquiring malignant phenotypes [61]. There was a sharp reduction in the expression of cell cycle regulatory genes, including ATM and ATR, in MCF10A-1 compared to MCF10A-2. Although these kinases are known to phosphorylate and regulate p53 function, we found that p53 activity was not altered in MCF10A-1. Strikingly, depletion of ATM alone was sufficient to convert MCF10A-2 cells from SATB1-resistant to SATB1-responsive cells, as indicated by the acquisition of malignant phenotypes upon overexpression of the protein. This phenomenon was observed also for two additional non-malignant immortalized breast epithelial cell lines, 184A1 and 184B5. Such selectivity for SATB1 activity suggests that reduced ATM level serves as a molecular determinant for SATB1 tumor-inducing activity in non-malignant cells. Interestingly, we also found that prolonged culture resulted in the phenotypic drift of MCF10A cells, leading to the reduction of ATM levels, and thereby, converting the cells from SATB1-resistant to SATB1-responsive types. Our results are consistent with a recent report showing that ATM depletion promotes cell proliferation, transformed phenotypes (e.g., morphology, invasiveness in culture) and genomic instability in MCF10A and MCF12 cells after prolonged culture [58]. Nevertheless, even after >20 passages, these ATM-depleted cells were only able to form dysplastic lesions and unable to form malignant tumors in immunodeficient mice [58], suggesting that an additional event must occur before adopting a malignant phenotype.

Reduced ATM expression was found not only in cancer cell lines but also in non-malignant MCF10A cells during passage in culture. This decline in ATM expression during repeated subculture may mimic an early stage in the progression of normal mammary epithelial cells towards malignancy *in vivo*. Decreased *ATM* expression is frequently found in breast tumors, but not in premalignant lesions, and is associated with poor patients' prognosis [62]. A premalignant gene expression signature that exhibits reduced ATM expression might be a prerequisite for SATB1 to induce cancer phenotypes. Conversely, high ATM expression could circumvent the tumorigenic effect of SATB1 expression. In support of this possibility, we found that SATB1 overexpression in normal or immortalized (e.g. MCF10A-2) human mammary epithelial cells prevented them from long term passage in culture, possibly due to abnormal cytokinesis and impaired cell cycle progression of these cells (unpublished results). We observed that MCF10A-1, but not MCF10A-2, had an impaired G2/M checkpoint. Since ATM plays a role in the mitotic checkpoint of the cell cycle [55], it is possible that low levels of ATM enable the survival of SATB1-overexpressing cells, allowing them to form aggressive tumors. The important functional link between ATM and SATB1 must be investigated in depth in the future. Furthermore, a number of genes involved in G2/M checkpoint and DNA double-strand break repair, including BRCA2, are significantly reduced in MCF10A-1, suggesting their relevance to the observed defect in G2/M arrest [63], [64]. Conversely, there are several genes, including SNCG, whose expression levels are significantly upregulated in MCF10A-1 (Fig. 4B). SNCG is involved breast cancer progression [65], [66] and implicated in the mitotic checkpoint through interaction with BubR1 [67]. Whether these genes also contribute to the premalignant gene expression signature required for SATB1 oncogenic activity will warrant further investigation.

Our data show that once non-malignant cells gain a gene expression signature permissive for SATB1-mediated oncogenesis, SATB1 can induce malignant phenotypes. Further, once SATB1 expression reaches a threshold level, there is a positive correlation between the SATB1 level and metastatic potential of cancer cells. In addition, altering the SATB1 level alone is sufficient to switch cells between highly metastatic and non-metastatic states. Interestingly, even a modest reduction in the SATB1 level (70%) abrogated lung metastasis, with no major reduction in tumor formation. This observation suggests that lung metastasis requires a significantly higher level of SATB1 than the formation of primary tumor. In sum, our results demonstrate a strong oncogenic role for SATB1 in immortalized cells under a permissive gene expression profile and uncover a tumor suppressive role for ATM in preventing SATB1-mediated oncogenesis. These data further suggest that SATB1 might serve as a novel and effective therapeutic target for treating invasive and metastatic breast cancers.

## Methods

### Ethics Statement

All animal work was done following Institutional Animal Care and Use Committee guidelines. The protocol was approved by the Animal Welfare and Research Committee (Permit number: 86R-12508-1109) of the Lawrence Berkeley National Laboratory, an accredited Association for Assessment and Accreditation of Laboratory Animal Care institution.

### Cell culture

MCF10A (MCF10A-2) cells were obtained from the American Type Culture Collection (ATCC) and maintained for <15 passages as described [32], [68]. The MCF10A (MCF10A-1) cells, obtained originally from ATCC, were maintained for >50 passages in culture medium containing fetal bovine serum, instead of horse serum. MCF10A-NeoT and MCF10A-CA1d (CA1d) cells were obtained from the Barbara Ann Karmanos Center and maintained in culture as described [43]. 184A1 and 184B5 cells were generously provided by Dr. Martha Stampfer from the Human Mammary Epithelial Cell Bank at Lawrence Berkeley National Laboratory and were maintained as described [33]–[35]. The retroviral packaging cell line Phoenix (Orbigen) was maintained according to the manufacturer's instructions and used to produce retroviruses that contain a vesicular stomatitis virus G (VSVG) protein. The lentivirus-packaging cell line, 293FT (Invitrogen), was maintained in G418 (500 µg/ml). BT549, BT549 (SATB1 shRNA), MDA-MD-231 and cloned SATB1 shRNA transfected cells (shRNA 1 and 2) were prepared and maintained as described [5]. Protein and RNA samples were prepared from subconfluent cells in the exponential phase of growth.

### Analysis of mRNA and protein expression

Total RNA was purified using TRI reagent (Sigma) and the RNeasy kit (Qiagen). Two µg of each RNA sample was reverse transcribed using the Superscript II RNase H First-Strand Synthesis system (Invitrogen). cDNAs were analyzed in triplicate using an ABI 7500 Fast Real-Time PCR System (Applied Biosystem). The following primers were used for RT-PCR analysis of 1) SATB1 expression: 5′-TGCAAA GGTTGCAGCAACCAAAAGC (forward) and 5′-AACATGGATAATGTGGGGCGGCCT (reverse) 3) MDM2 expression: 5′-ACCTCACAGATTCCAGCTTCG (forward) and 5′-TTTC ATAGTATAAGTGTCTTTT (reverse), and 4) p21 expression: 5′-GGGGAAGGGACACACAAGAAGA (forward) and 5′-AATGAACTGGGGAGGGATGG (reverse). For multi-gene expression analysis, we used Superarrays (Qiagen; cancer progression, cell cycle and p53 pathways). We prepared RNA and cDNA according to manufacturer's instructions, and data analysis was done using the software provided by the manufacturer. Protein levels were assessed by immunoblotting using cell lysates (40–60 µg) in buffer (20 mM HEPES (pH 7.9), 25% glycerol, 0.5N NaCl, 1 mM EDTA, 1% NP-40, 0.5 mM dithiothreitol, 0.1% deoxycholate) containing protease inhibitors (Roche). We used antibodies against GAPDH (Milipore), SATB1 (Epitomics), SATB1, E-cadherin, fibronectin, ß-catenin (clone 14), integrin α6 (CD49f) (BD Biosciences), ERBB2, vimentin (Lab Vision Corp), β-actin, α-Tubulin (Sigma), and ATM (Genetex).

### Overexpression of SATB1

The human SATB1 cDNA was subcloned into either the pLXSN retroviral vector (Clontech) or pCDF1 lentiviral vector (Systems Biosciences). pCDF1-SATB1 was used for the MCF10A-NeoT and progression cell line CA1d, whereas pLXSN-SATB1 was used for MCF10A-1, MCF10A-2, 184A1, and 184B5 cells. Both viral transduction methods were confirmed to give similar results. The pLXSN-SATB1 construct was transfected into Phoenix packaging cells using FuGene HD (Roche). Virus-containing supernatants were collected after 24–60 h, and titered. Viral media was added twice with a 24 h interval to 50% confluent cells in the presence of 8 µg/ml polybrene (Sigma). Forty-eight hours later, cells were given 800 µg/ml of G418 to select for stably infected clones. Lentivirus production and transduction were conducted according to the System Biosciences guidelines. Briefly, lentivirus vector and packaging plasmid mix were transfected into 293FT cells using lipofectamine 2000 (Invitrogen). After 48 h, medium was harvested, filtered and used to infect target cells with the addition of polybrene (10 µg/ml). After 24 h, the medium was replaced with fresh medium. At 72 h post-infection, puromycin (0.5 µg/ml) was added for selection and maintained throughout the culturing period.

### SATB1- and/or ATM-knockdown cells

To prepare SATB1 knockdown cells, cells were transfected with the pSUPER-puro (Oligoengine) construct harboring oligoduplexes prepared from shRNA_2176_ 5′-GGATTTGGAAGAGAGTGTC and selected with 1.5 µg/ml puromycin [5]. The sequences used for ATM silencing were 5′-AACATACTACTCAAAGACATT (at 812), 5′-GCACCAGTCCAGTATTGGCTT (at 2404) [69] (or 5′-GCAGAGTCAATCATAGA (at 3653) [58]. The sequences were also cloned into pSUPER-retro-puro (Oligoengine). All sequences were confirmed on both strands. The constructs were transfected into MCF10A-1, MCF10A-2, 184A1 and 184B5 cells, which were selected in puromycin. The levels of ATM were confirmed by qRT-PCR and immunoblot analysis. As the negative control, we used a sequence that targets the EGFP cDNA (5′-GAAGCAGCACGACTTCTTC), which was cloned into either pSUPER-puro or pSUPER-retro-puro.

### Three-Dimensional (3D) Morphological analysis

Three-dimensional morphological analysis was done by seeding approximately 5000 cells per well in 24-well plates. Each well was coated with 150 µl of growth factor reduced Matrigel (BD Biosciences) and maintained as described [70]. Phase contrast images were taken after 3 and 6 days using the EVOS imaging system (AMG).

### Chemoinvasion assay

Assays were performed in 24-well chemotaxis plates with an 8µm polycarbonate filter coated with growth-factor-reduced Matrigel (BD Biosciences) diluted to 15%. The cells were resuspended in serum-free medium (5×10^4^ cells per well) and added to the upper chamber. Conditioned media from NIH3T3 fibroblast cultures were placed in the lower chambers as a chemo-attractant. The chambers were incubated for 20 h at 37°C with 5% CO_2_; experiments were performed in triplicate. Cells that migrated to the undersides of the filter were fixed in cold methanol and stained with crystal violet. The migrated cells were counted by light microscopy. S.e.m. values were determined for each sample.

### Cell proliferation assay

Cell proliferation was measured by seeding approximately 3×10^4^ cells on plastic culture dishes, or 1×10^4^ cells on Matrigel-coated 60 mm dishes. At the indicated time points, cells on Matrigel were incubated with dispase (BD Biosciences) for 2 h at 37°C and then trypsin for 10 min. Cells were counted using a Coulter counter. Trypan blue exclusion analysis indicated that 99–100% of the cells were viable.

### Soft agar colony formation assay

Cells (1×10^4^) were suspended in DMEM containing 5% FBS with 0.3% agarose and layered on atop 0.5% agarose in DMEM in 60-mm plates. Cultures were maintained for 25 days. Colonies were fixed and stained with crystal violet. Colonies greater than 20 µm in diameter were scored as positive. Each experiment was done in triplicate.

### Immunofluorescence analysis

3D cultures were prepared by seeding approximately 3000 cells per well on Matrigel-coated 8 well chamber slides. The cells were fixed in 4% paraformaldehyde, permeabilized in 0.5% Triton-X100 and blocked in 10% normal goat serum. Focal adhesion complexes were detected with anti-ß catenin (clone 14), anti-fibronectin, anti-E-cadherin, and anti-integrin α6 (CD49f) (BD Biosciences). F-actin was detected by fluorescent phalloidin (Invitrogen). Fixed and permeabilized cells were incubated with primary antibodies overnight at 4°C, followed by Alexa Fluor 488 and/or Alexa Fluor 594 secondary antibodies (Invitrogen). Images were collected using a Delta Vision microscope and processed with SoftWoRx software (Applied Precision).

### Primary tumor cell culture

Tumors formed by injection of SATB1 overexpressing MCF10A-1 (single clone clone-1) cells into nude mice were isolated and subjected to enzymatic dissociation by 0.2% collagenase type-2 in DMEM for 2 h at 37°C. The resulting tumor cell line (clone-1-TUM) was maintained in DMEM/F12(1∶1) medium containing G418 (800 µg/ml).

### Analysis of tumour growth and experimental metastasis

Four to seven female NCR athymic mice (6–7 weeks old, Taconic) were injected with parental cells, vector control cells, a pool of SATB1-overexpressing cells (pLXSN-SATB1), a single SATB1-overexpressing clone (clone-1), clone1-1-TUM (vector control), and clone-1-TUM (shRNA-SATB1). Cells (5×10^5^ with Matrigel at 7 µg/ml in PBS in a volume of 150 µl) were injected into the fourth mammary fat pad. Tumour growth was monitored using vernier calipers for 6–7 weeks after injection. For metastasis analysis, 1×10^6^ cells in 150 µl PBS were injected intravenously via the lateral tail vein. At 9–10 weeks after injection, the mice were sacrificed and the lungs removed and fixed in 10% formalin. The number of surface metastases per lung was determined under a dissecting microscope.

### SNP Array and DNA Copy Number Analysis

DNA extracted from MCF10A-1 and MCF10A-2 cells was labeled and hybridized to the Affymetrix Genome-Wide Human SNP Array 6.0 for DNA copy number analysis, which can genotype over 900K SNP markers across the genome. The Affymetrix Genotyping Console Software was used to make genotype calls and determine copy number. The default settings were used for all analyses. All genotype calls that were in disagreement between the two samples were identified. SNPs for which either one of both samples failed to yield a genotype call were excluded. Percent similarity was calculated as follows: D/N * 100, where D  =  the number of SNPs with differing genotypes in the two samples, and N  =  total number of SNPs measured. Copy number similarity was assessed by plotting the smoothed copy number value for each sample. The Pearson correlation was determined between the two sets of copy number values to assess similarity between the samples.

### Cell cycle analysis

For G2/M checkpoint analysis, 4×10^5^ cells/well were plated onto 6-well culture dishes. Cells were given medium supplemented with 2.5 mM hydroxyurea (HU; Sigma) for 24 h. Cells were washed 3 times with culture medium, and then continuously incubated in culture media containing 500 nM paclitaxel (Sigma). Cells were collected 0, 18, 22 and 28 h later and fixed with 5 ml ice cold 70% ethanol for 1 h at 4 C. Cells were stained with 50 µg/ml propidium iodine/50 µg/ml RNase/PBS for 30 min in the dark. Cell cycle analysis was performed using the Guava system (Millipore) and analyzed using FlowJo software.

## Supporting Information

Figure S1 **A**) Immunoblot showing the expression levels of SATB1 protein before and after viral transduction of SATB1 in MCF10A-neoT and CA1d cell lines. ß-actin was used as an internal loading control. **B**) Colony morphologies of control and SATB1 overexpressing MCF10A-neoT and CA1d cells (drug-selected pooled cell populations), and BT549 control and SATB1 depleted (SATB1-shRNA) cells were compared in 3D Matrix on-top cultures. MCF10A images were captured six days after plating with Phase 1 at 20X magnification, and BT549 images were captured three days after plating at 10X magnification. In MCF10A progression cell series, SATB1 expression induced predominant network-like structures. Scale bars, 100 µm. **C**) (Top) Immunoblot showing the expression levels of MCF10A-1 pLXSN-SATB1 single clones clone-1 and clone-2. α-tubulin was used as an internal loading control. (Bottom) Colony morphologies of clone-1 and clone-2 in 3D Matrix on-top cultures six days after plating. The images were captured with Phase 1 at 20X magnification. Scale bars, 100 µm. **D**) Immunostaining for ß-catenin, fibronectin, E-cadherin and DAPI using MDA-MB-231 derivatives (vector control and SATB1-shRNA1) and MCF10A-1 derivatives (vector control and clone-1) grown on 2D culture. Images were captured by fluorescence microscopy at 20x magnification. Scale bars, 15 µm.
(PDF)

Figure S2 **A**) Average weight (gram) of tumors isolated 40 days after mammary fat pad injection with MCF10A-1 vector control, pLXSN-SATB1 pool, and clone-1. (n = 4 to 5 mice per group). Error bars indicate s.e.m. **B**) Incidence of total palpable tumors in clone-1-TUM and clone-1-TUM(SATB1-shRNA) after mammary fat pad injection. This indicates a delay in onset of tumor formation for clone-1-TUM (SATB1-shRNA) cells compared to clone-1-TUM during the first week.
(PDF)

Figure S3 **A**) The graphical analysis comparing Chromosomes 9 and 13 of MCF10A-1 and MCF10A-2 was generated using the R package DNA copy to segment the log2 ratio values computed from the Affymetrix implementation of Birdseed. Each dot represents the log2 copy number for one SNP. The red line represents the segmented copy number value. The segments represent genomic regions of similar copy number. **B**) (Top) p53 activation is comparable in MCF10A-1 and MCF10A-2 cells, as shown by phosphorylation of serine 15 upon 12Gy x-ray irradiation. (Bottom) In response to 12Gy x-ray irradiation, p21 and MDM2 are induced for both MCF10A-1 and MCF10A-2 cells, strongly suggesting that p53 function is normal in these cells. mRNA levels are measured by qPCR and normalized against average levels of GAPDH, TBP, and L32 control genes. **C**) Expression analysis of genes associated in P53 pathway in MCF10A-1 and MCF10A-2. Genes are categorized according to their functional gene groupings. Relative fold levels of gene expression in MCF10A-1 (blue bar) against MCF10A-2 (red dotted line) are shown. Each of gene expression level was normalized to the level of GAPDH.
(PDF)

Figure S4 (Top) Schematic diagram of M phase cell cycle checkpoint analysis. Cells were synchronized with 2.5 mM HU for 24 h, followed by removal of HU and continuous incubation in media containing 500 nM paclitaxel. After 0, 18, 22, and 28 h incubation with paclitaxel, cells were analyzed for cell cycle distribution. (Top two rows) Cell cycle profiles of MCF10A-1 treated with HU and HU+paclitaxel. (Bottom two rows) Cell cycle profiles of MCF10A-2 treated with HU and HU+pacltaxel. At 22 h, percentages are shown for each cell cycle gate (Apoptosis, G1, S, and G2/M) to demonstrate a defect in the phase checkpoint of MCF10A-1. Cell cycle profiles are representative of three experiments.
(PDF)

Figure S5 **A**) Quantitative RT-PCR analysis for ATM expression normalized to GAPDH. RNA and cDNA were prepared from parental cell lines MCF10A-1, MCF10A-2, MCF10A-1_early (fresh culture from frozen stocks prepared after few passages), MCF10A-1_(90D) (MCF10A-1 cultured for an additional 3 months), 184A1, 1845B5, MCF10A-neoT, CA1d, BT549 and MDA-MB-231. **B**) Western blot showing expression levels of ATM in MCF10A-2, MCF10A-1_early, MCF10A-1 and MCF10A-1_(90D). **C**) Quantitative RT-PCR analysis for SATB1 expression relative to GAPDH in parental cell lines of MCF10A-1, MCF10A-2, MCF10A-1_early, MCF10A-1_(90D), 184A1, 1845B5, MCF10A-NeoT, CA1d, BT549 and MDA-MB-231. **D**) (Left) Immunoblot showing the SATB1 expression levels of MCF10A-1_early pLXSN-SATB1. α-tubulin was used as an internal loading control. (Right) Colony morphologies of control and SATB1 overexpressing MCF10A-1, MCF10A-1_early, and MCF10A-2 cells (drug-selected pooled cell populations) were compared in 3D Matrix on-top cultures. The images were captured with Phase 1 at 20X magnification. Scale bars, 100 µm. **E**) Invasion assay of MCF10A-1, MCF10A-2, MCF10A-1_early, MCF10A-1_(90D), MCF10A-neoT and CA1d cell lines before and after SATB1 overexpression. Parental cells lines are shown in blue; SATB1 overexpressing cells are shown in pink. Error bars indicate s.e.m., n = 3 experiments.
(PDF)
