# Supplementary figures and images for: ATM Suppresses SATB1-Induced Malignant Progression in Breast Epithelial Cells
Source: PLoS One. 2012 Dec 10;7(12):e51786. doi: 10.1371/journal.pone.0051786 (PMC3519734; doi:10.1371/journal.pone.0051786)

# Supplementary Figure 1

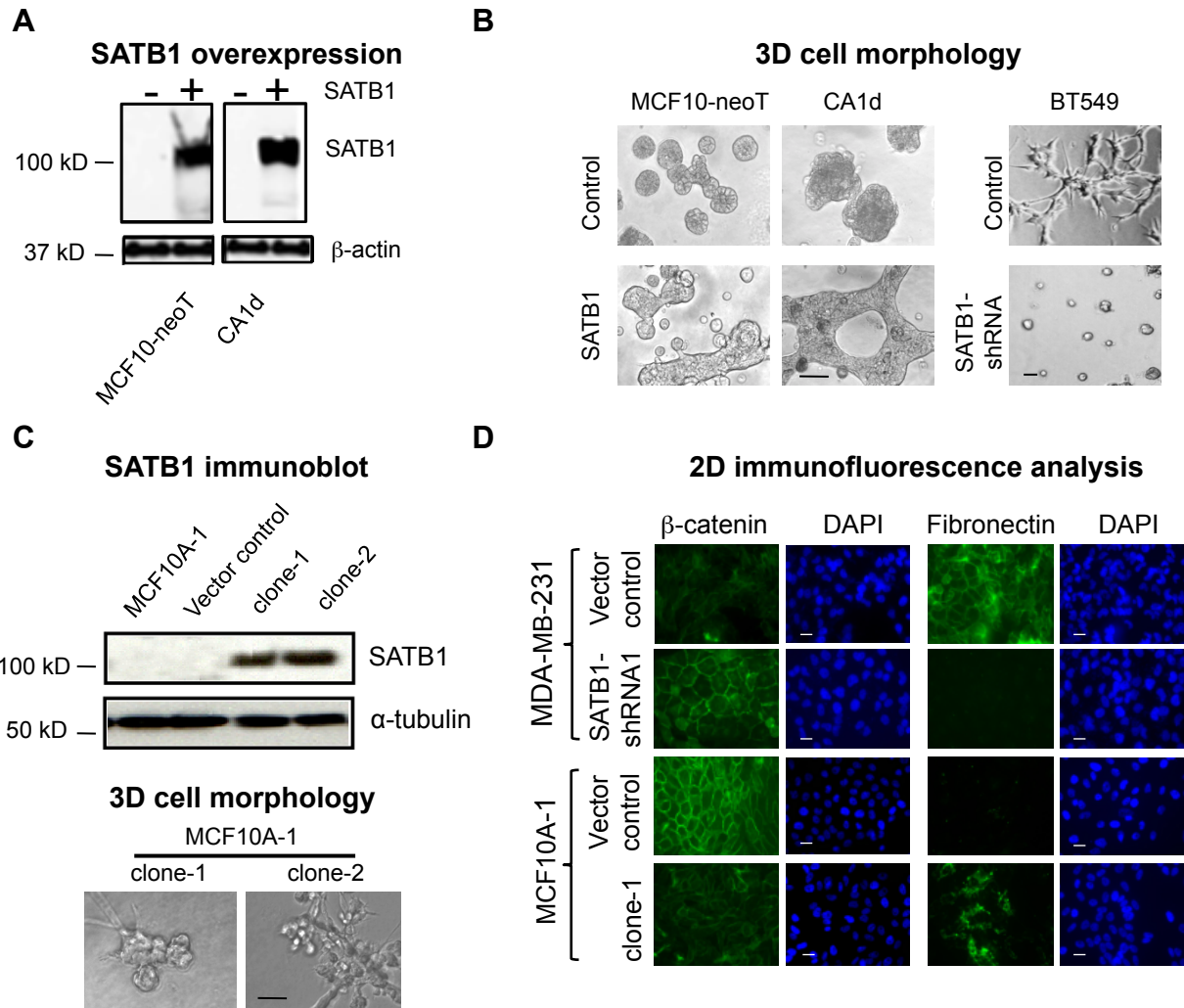

Supplement: Figure S1 — A) Immunoblot showing the expression levels of SATB1 protein before and after viral transduction of SATB1 in MCF10A-neoT and CA1d cell lines. ß-actin was used as an internal loading control. B) Colony morphologies of control and SATB1 overexpressing MCF10A-neoT and CA1d cells (drug-selected pooled cell populations), and BT549 control and SATB1 depleted (SATB1-shRNA) cells were compared in 3D Matrix on-top cultures. MCF10A images were captured six days after plating with Phase 1 at 20X magnification, and BT549 images were captured three days after plating at 10X magnification. In MCF10A progression cell series, SATB1 expression induced predominant network-like structures. Scale bars, 100 µm. C) (Top) Immunoblot showing the expression levels of MCF10A-1 pLXSN-SATB1 single clones clone-1 and clone-2. α-tubulin was used as an internal loading control. (Bottom) Colony morphologies of clone-1 and clone-2 in 3D Matrix on-top cultures six days after plating. The images were captured with Phase 1 at 20X magnification. Scale bars, 100 µm. D) Immunostaining for ß-catenin, fibronectin, E-cadherin and DAPI using MDA-MB-231 derivatives (vector control and SATB1-shRNA1) and MCF10A-1 derivatives (vector control and clone-1) grown on 2D culture. Images were captured by fluorescence microscopy at 20x magnification. Scale bars, 15 µm. (PDF) [file pone.0051786.s001.pdf]

# Supplementary Figure 2

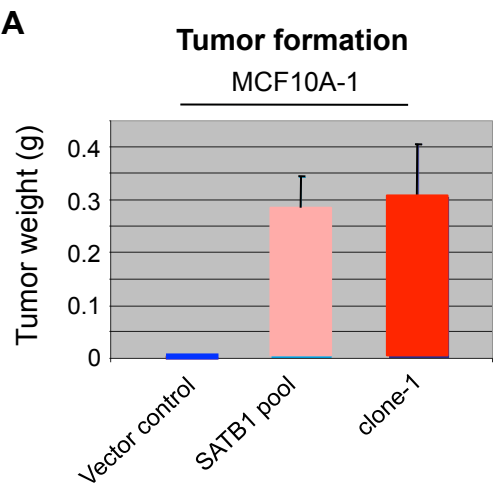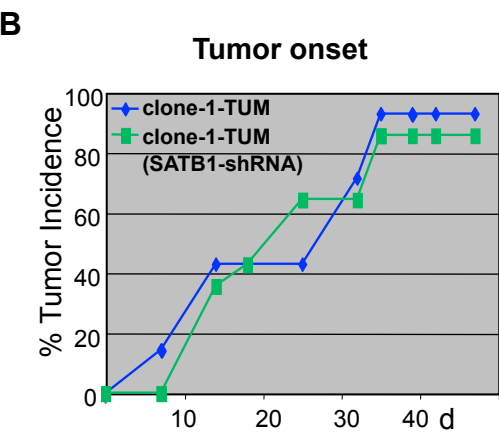

Supplement: Figure S2 — A) Average weight (gram) of tumors isolated 40 days after mammary fat pad injection with MCF10A-1 vector control, pLXSN-SATB1 pool, and clone-1. (n = 4 to 5 mice per group). Error bars indicate s.e.m. B) Incidence of total palpable tumors in clone-1-TUM and clone-1-TUM(SATB1-shRNA) after mammary fat pad injection. This indicates a delay in onset of tumor formation for clone-1-TUM (SATB1-shRNA) cells compared to clone-1-TUM during the first week. (PDF) [file pone.0051786.s002.pdf]

# Supplementary Figure 3

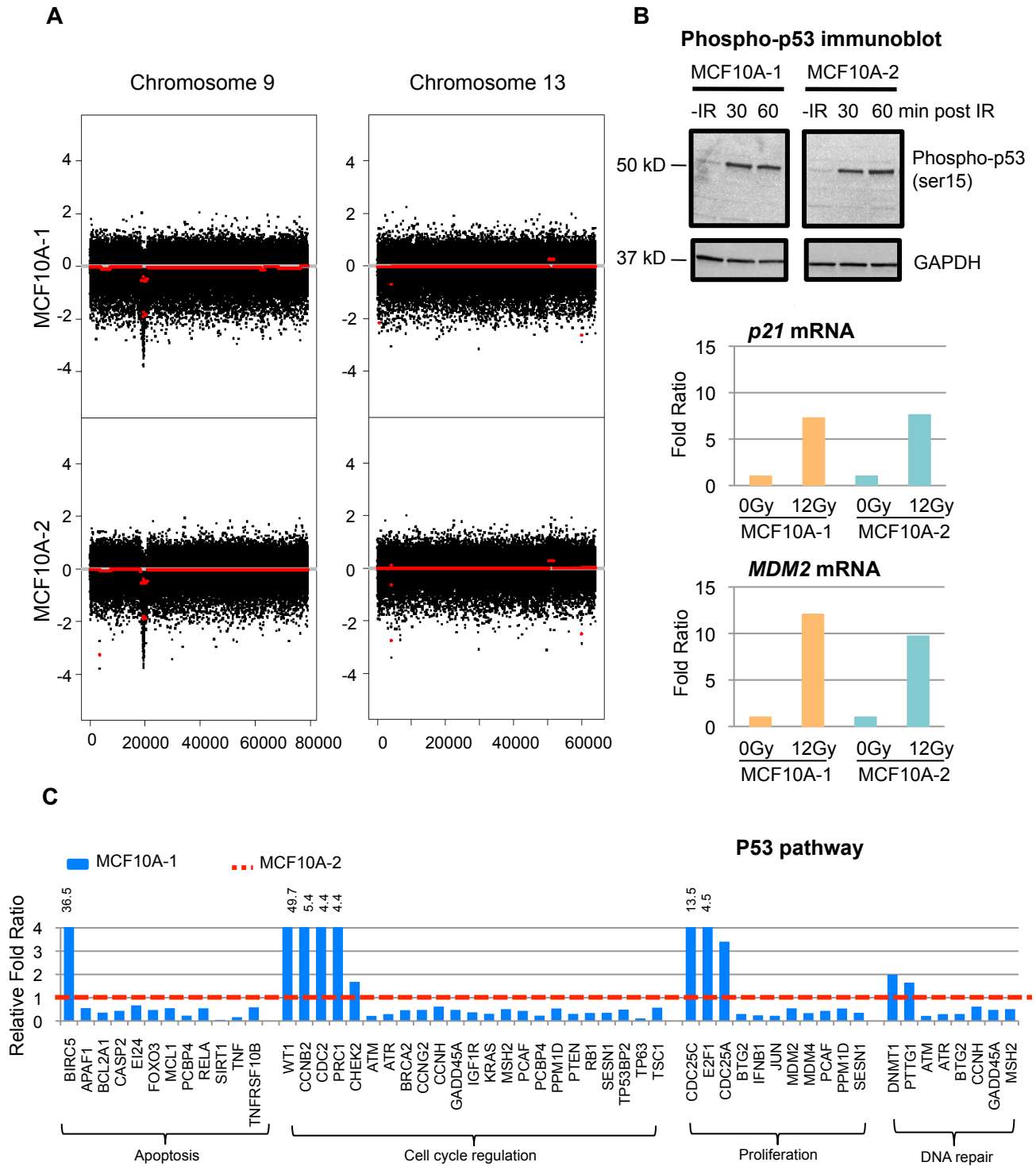

Supplement: Figure S3 — A) The graphical analysis comparing Chromosomes 9 and 13 of MCF10A-1 and MCF10A-2 was generated using the R package DNA copy to segment the log2 ratio values computed from the Affymetrix implementation of Birdseed. Each dot represents the log2 copy number for one SNP. The red line represents the segmented copy number value. The segments represent genomic regions of similar copy number. B) (Top) p53 activation is comparable in MCF10A-1 and MCF10A-2 cells, as shown by phosphorylation of serine 15 upon 12Gy x-ray irradiation. (Bottom) In response to 12Gy x-ray irradiation, p21 and MDM2 are induced for both MCF10A-1 and MCF10A-2 cells, strongly suggesting that p53 function is normal in these cells. mRNA levels are measured by qPCR and normalized against average levels of GAPDH, TBP, and L32 control genes. C) Expression analysis of genes associated in P53 pathway in MCF10A-1 and MCF10A-2. Genes are categorized according to their functional gene groupings. Relative fold levels of gene expression in MCF10A-1 (blue bar) against MCF10A-2 (red dotted line) are shown. Each of gene expression level was normalized to the level of GAPDH. (PDF) [file pone.0051786.s003.pdf]

# Supplementary Figure 4

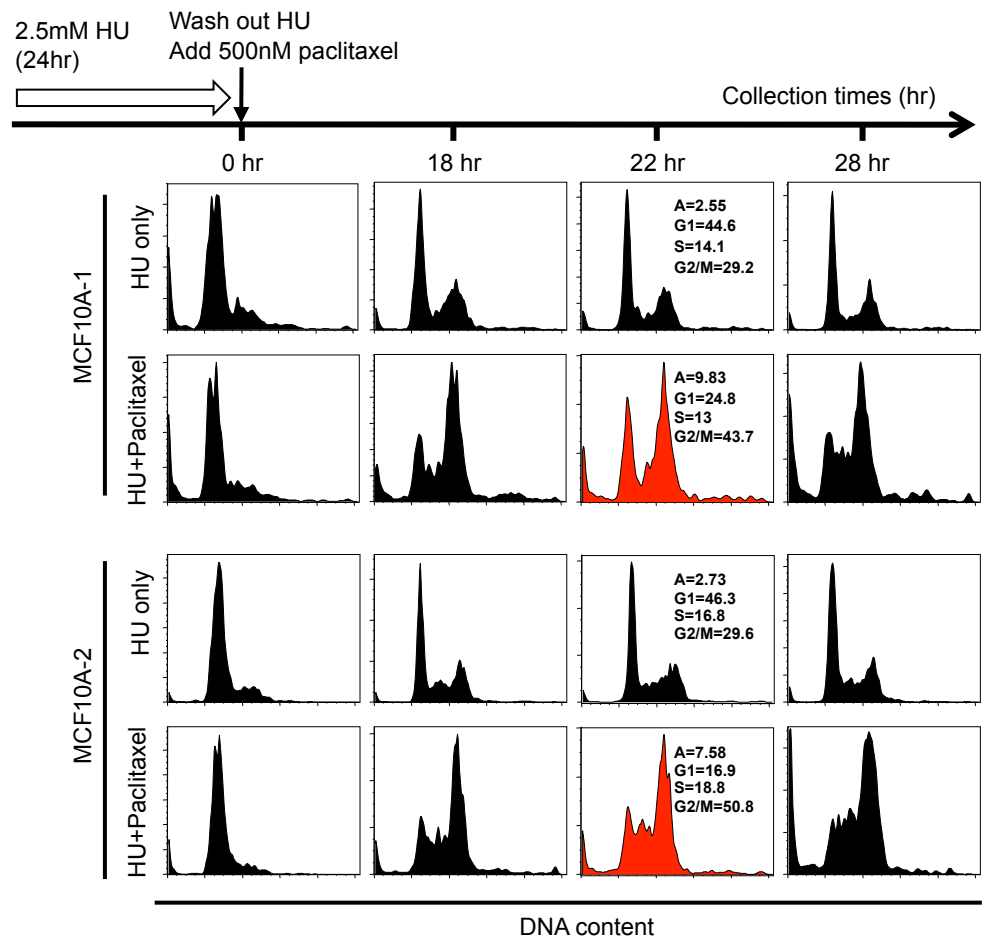

Supplement: Figure S4 — (Top) Schematic diagram of M phase cell cycle checkpoint analysis. Cells were synchronized with 2.5 mM HU for 24 h, followed by removal of HU and continuous incubation in media containing 500 nM paclitaxel. After 0, 18, 22, and 28 h incubation with paclitaxel, cells were analyzed for cell cycle distribution. (Top two rows) Cell cycle profiles of MCF10A-1 treated with HU and HU+paclitaxel. (Bottom two rows) Cell cycle profiles of MCF10A-2 treated with HU and HU+pacltaxel. At 22 h, percentages are shown for each cell cycle gate (Apoptosis, G1, S, and G2/M) to demonstrate a defect in the phase checkpoint of MCF10A-1. Cell cycle profiles are representative of three experiments. (PDF) [file pone.0051786.s004.pdf]

## Supplementary Figure 5

**A**

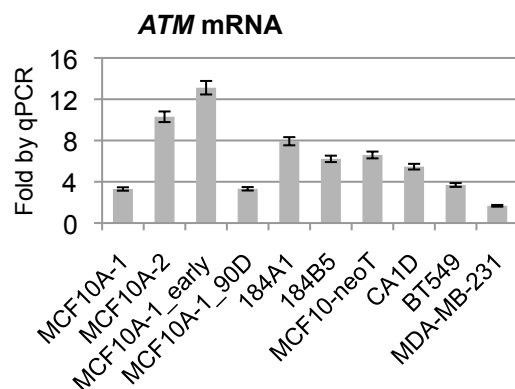

**B**

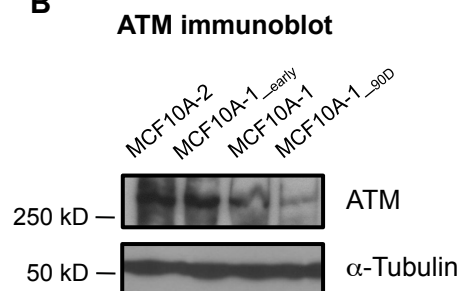

**C**

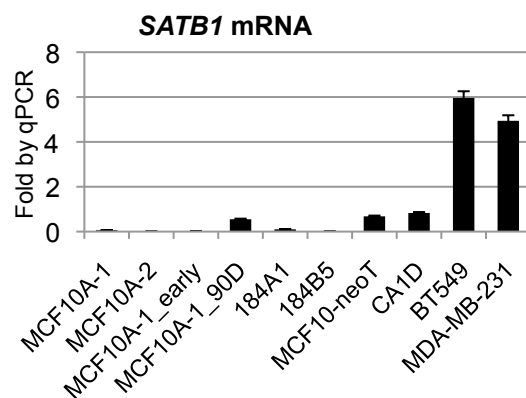

**D**

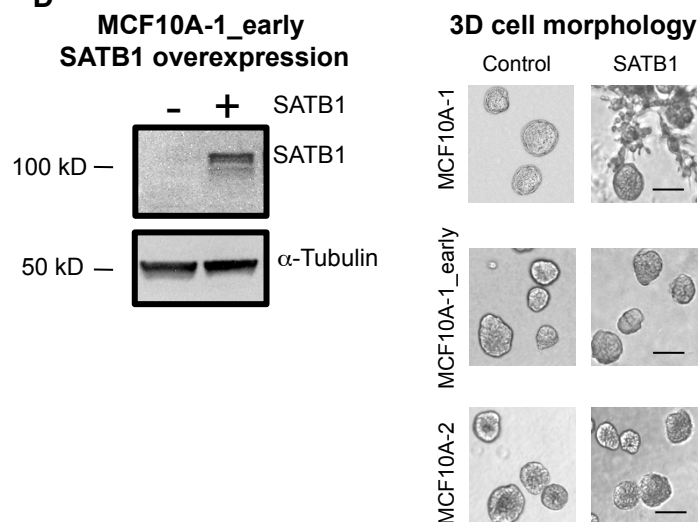

**E**

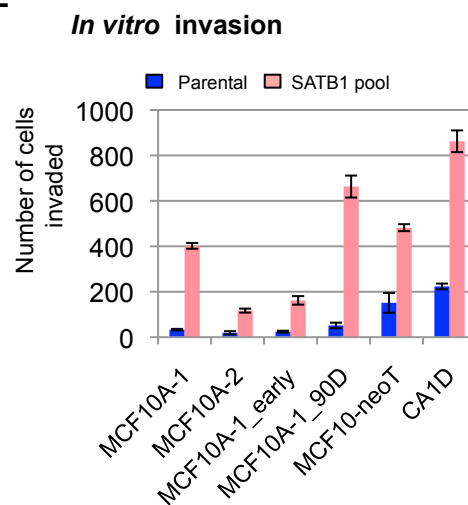

Supplement: Figure S5 — A) Quantitative RT-PCR analysis for ATM expression normalized to GAPDH. RNA and cDNA were prepared from parental cell lines MCF10A-1, MCF10A-2, MCF10A-1_early (fresh culture from frozen stocks prepared after few passages), MCF10A-1_(90D) (MCF10A-1 cultured for an additional 3 months), 184A1, 1845B5, MCF10A-neoT, CA1d, BT549 and MDA-MB-231. B) Western blot showing expression levels of ATM in MCF10A-2, MCF10A-1_early, MCF10A-1 and MCF10A-1_(90D). C) Quantitative RT-PCR analysis for SATB1 expression relative to GAPDH in parental cell lines of MCF10A-1, MCF10A-2, MCF10A-1_early, MCF10A-1_(90D), 184A1, 1845B5, MCF10A-NeoT, CA1d, BT549 and MDA-MB-231. D) (Left) Immunoblot showing the SATB1 expression levels of MCF10A-1_early pLXSN-SATB1. α-tubulin was used as an internal loading control. (Right) Colony morphologies of control and SATB1 overexpressing MCF10A-1, MCF10A-1_early, and MCF10A-2 cells (drug-selected pooled cell populations) were compared in 3D Matrix on-top cultures. The images were captured with Phase 1 at 20X magnification. Scale bars, 100 µm. E) Invasion assay of MCF10A-1, MCF10A-2, MCF10A-1_early, MCF10A-1_(90D), MCF10A-neoT and CA1d cell lines before and after SATB1 overexpression. Parental cells lines are shown in blue; SATB1 overexpressing cells are shown in pink. Error bars indicate s.e.m., n = 3 experiments. (PDF) [file pone.0051786.s005.pdf]
